# Carbon Monoxide Blocks Lipopolysaccharide-Induced Gene Expression by Interfering with Proximal TLR4 to NF-κB Signal Transduction in Human Monocytes

**DOI:** 10.1371/journal.pone.0008139

**Published:** 2009-12-02

**Authors:** Maneesha Chhikara, Shuibang Wang, Steven J. Kern, Gabriela A. Ferreyra, Jennifer J. Barb, Peter J. Munson, Robert L. Danner

**Affiliations:** 1 Critical Care Medicine Department, Clinical Center, National Institutes of Health, Bethesda, Maryland, United States of America; 2 Mathematical and Statistical Computing Laboratory, Center for Information Technology, National Institutes of Health, Bethesda, Maryland, United States of America; Cairo University, Egypt

## Abstract

Carbon monoxide (CO) is an endogenous messenger that suppresses inflammation, modulates apoptosis and promotes vascular remodeling. Here, microarrays were employed to globally characterize the CO (250 ppm) suppression of early (1 h) LPS-induced inflammation in human monocytic THP-1 cells. CO suppressed 79 of 101 immediate-early genes induced by LPS; 19% (15/79) were transcription factors and most others were cytokines, chemokines and immune response genes. The prototypic effects of CO on transcription and protein production occurred early but decreased rapidly. CO activated p38 MAPK, ERK1/2 and Akt and caused an early and transitory delay in LPS-induced JNK activation. However, selective inhibitors of these kinases failed to block CO suppression of LPS-induced IL-1β, an inflammation marker. Of CO-suppressed genes, 81% (64/79) were found to have promoters with putative NF-κB binding sites. CO was subsequently shown to block LPS-induced phosphorylation and degradation of IκBα in human monocytes, thereby inhibiting NF-κB signal transduction. CO broadly suppresses the initial inflammatory response of human monocytes to LPS by reshaping proximal events in TLR4 signal transduction such as stress kinase responses and early NF-κB activation. These rapid, but transient effects of CO may have therapeutic applications in acute pulmonary and vascular injury.

## Introduction

Carbon monoxide (CO), an endogenous messenger generated by heme oxygenase-1 (HO-1) [1], has cytoprotective [2], [3], anti-proliferative [4]–[6], and anti-inflammatory effects [7]–[10] that indicate a potential for clinical applications. In animal models, CO itself or CO-releasing molecules (CORMs) have demonstrated benefits in ischemia/reperfusion injury [11]–[13], pulmonary inflammation [8], [14], [15], and sepsis [10], [16]. However, the intermediary targets of CO signaling and mechanisms of its therapeutic effects are not entirely clear. Unlike nitric oxide (NO^•^), CO only weakly activates soluble guanylate cyclase [4] and therefore cGMP may be less important as a second messenger.

The anti-inflammatory effects of CO have been extensively investigated in rodents. CO was shown in mice to activate p38 MAPK and induce IL-10, thereby downregulating proinflammatory cytokines such as TNF-α, IL-1β and MIP-1β [7]. A study in rats reported that CO blocks inflammatory responses through cGMP-dependent inhibition of ERK and suppression of early growth factor (EGR)-1, an immediate-early transcription factor [17]. The anti-inflammatory effects of CO have also been associated with an early delay in LPS-induced JNK activation and subsequent impairment of AP-1 signaling [18]. Other investigations have emphasized CO interference with NF-κB signal transduction [16], [19]–[22]. Alternatively, CO has been shown to block apoptosis by activating NF-κB [23], [24]. Release of oxygen radicals from mitochondria was identified as a proximal signaling event in CO activation of NF-κB [24], as well as CO effects on p38 MAPK [25], peroxisome proliferator-activated receptor gamma (PPARγ) [26] and hypoxia-inducible factor (HIF-α) [27]. Conversely, other work has associated the anti-inflammatory activity of CO with NADPH oxidase inhibition and decreased oxygen radical generation [20], [28]. Collectively, these findings in rodents suggest a complex, compartmentalized pattern of signaling that appears to be highly dependent on experimental conditions and timing.

Studies using human cells are more limited, but also demonstrate a range of effects and similarly indicate that CO signal transduction may be context dependent. In A549 cells, a human pulmonary epithelial line, CO gas (250 ppm) inhibited IL-17-induced activation of ERK1/2 and had no effect on p38 MAPK or JNK[29]. Somewhat differently, CO was shown in Caco-2 cells (an adenocarcinoma line), to decrease cytokine-induced activation of p38 MAPK, JNK and ERK1/2 as well as NF-κB, [21]. Another group also reported that CO suppressed NF-κB activation in LPS-challenged human umbilical vein endothelial cells (HUVEC), attenuating the induction of intercellular adhesion molecule-1 (ICAM-1) and nitric oxide synthase 2 (NOS2) expression [30]. Again suggesting that CO may decrease NAPDH oxidase function and ROS production, CO was found to dampen the respiratory burst in human neutrophils [31]. However, inhaled CO gas (500 ppm) failed to reduce LPS-induced inflammation in human volunteers, despite substantial anti-inflammatory effects in LPS-challenged mice [32]. Thus, human compared to rodent studies show both overlap and differences. Defining the mechanisms, kinetics and targets of CO signaling in human cells is essential to understanding its therapeutic potential.

The goals of this investigation were to identify the earliest gene targets of CO signaling in human monocytes and to examine its mechanisms. Oligonucleotide microarrays were used to globally characterize the effects of CO on Toll-like-receptor (TLR) 4-mediated gene regulation 1 h after LPS challenge in human THP-1 cells. Pivotal target genes and signal transduction events were subsequently verified in primary human monocytes. The predominant effect of CO was to suppress LPS-induced immediate-early genes including transcription factors, cytokines and chemokines. Suppression of gene transcription was rapid, but also transient. Several LPS-responsive stress kinase pathways, p38 MAPK, ERK1/2 and Akt, were activated by CO, while LPS-induced JNK phosphorylation was briefly delayed. However, kinase inhibitors failed to block CO-suppression of IL-1β, indicating that effects on these pathways were tangential rather than causative. Most of the genes suppressed by CO were associated with NF-κB regulation and CO was shown to interfere with an early step in LPS/TLR4 mediated NF-κB activation in human monocytes. The mechanism and kinetics of inflammatory response suppression by CO has implications for its use as a therapeutic strategy.

## Materials and Methods

### Reagents

*Salmonella minnesota* Re595 LPS was obtained from List Biologic (Campbell, CA). Cyclic nucleotide analogue, 8-bromo cGMP was purchased from Sigma Aldrich (St. Louis, MO). MAPK inhibitors: p38 (SB-203580), ERK (PD-98059), JNK Inhibitor II and Akt (LY-294002) were obtained from Calbiochem (San Diego, CA). Antibodies against phospho-IκB-α (5A5), p38 MAPK, ERK1/2, JNK, Akt and IL-1β were purchased from Cell Signaling Technology (San Jose, CA). Santa Cruz Biotechnology (Santa Cruz, CA) was the source of anti-IκB-α (C-21), anti-Tubulin and anti-Ah Receptor (C-18) antibodies, as well as double stranded NF-κB oligonucleotide consensus sequence and mutant NF-κB oligonucleotide. Anti-rabbit and anti-mouse IgG secondary antibodies were from Jackson ImmunoResearch Laboratories (West Grove, PA). RNeasy kits were obtained from Qiagen (Valencia, CA). ELISA kits for IL-1β and IL-8 were purchased from R&D Systems (Minneapolis, MN).

### Cell Culture

THP-1 cells (TIB-202), a human monocytic cell line, from American Type Culture Collection (ATCC, Manassas, VA) were cultured in RPMI-1640 complete medium (Invitrogen, Carlsbad, CA) with L-glutamine (2 mmol/L) containing 10% heat-inactivated fetal calf serum, 100 U/ml penicillin, 100 µg/ml streptomycin, and 50 µM β-mercaptoethanol (Sigma-Aldrich) at 37°C in a humidified atmosphere of 5% CO_2_. Human elutriated monocytes from healthy donors were obtained from the NIH Clinical Center Department of Transfusion Medicine (Bethesda, MD) using an IRB-approved protocol. Monocytes were then spun at 1500 rpm for 5 min and resuspended in Erythrocyte lysis buffer (Qiagen). Cell pellets were washed twice in PBS buffer, resuspended in RPMI 1640 complete medium and incubated overnight. More than 95% of these cells expressed CD14, as determined by flow cytometry using fluorescein isothiocyanate-labeled anti-human CD14 (BD Biosciences, San Jose, CA).

### LPS Challenge and Carbon Monoxide Exposure

LPS (1 µg/ml) was used as indicated to stimulate THP-1 cells or elutriated primary human monocytes. Incubated cells were exposed to CO at 250 ppm in special modular incubator chambers (Billups-Rothenberg Inc., CA) using premixed gas cylinders (Praxair, Bethlehem, PA) containing compressed air and 250 ppm CO supplemented with 5% CO_2_ for adjustment of pH. Final CO levels were monitored using a mini CO analyzer (MSA, Pittsburg, PA). CO exposure time was varied according to the experimental design. To ensure that gene suppression was not due to CO cytotoxicity, cell viability was verified by quantifying live cells using a nucleocounter (New Brunswick Scientific, Edison, NJ).

### Oligonucleotide Microarrays

Total RNA extracted from THP-1 cells was subjected to gene expression profiling using HGU133 plus 2.0 arrays according to manufacturer's protocols (Affymetrix, Santa Clara, CA). THP-1 cells (1×10^7^) incubated with or without LPS (1 µg/ml) in presence or absence of CO (250 ppm) were lysed with RLT buffer (Qiagen) and shredded (Qiashredder column). Total RNA was extracted using RNeasy Mini kit (Qiagen), following the manufacturer's instructions. Quality of total RNA was evaluated using RNA 6000 Nano LabChip (Agilent 2100 Bioanalyzer, Santa Clara, CA). All samples had intact 18S and 28S ribosomal RNA bands with RIN numbers from 8.8 to 9.4 and RNA 260/280 ratios between 1.9 and 2.0. Double-stranded cDNA was synthesized from total RNA (1.7 µg) and purified using a GeneChip Expression 3′- One-Cycle cDNA Synthesis kit (Affymetrix). Biotin labeled cRNA was made using an IVT GeneChip Expression 3′-Amplification kit (Affymetrix), fragmented and hybridized to microarrays following the manufacturer's directions. After staining with streptavidin-phycoerythrin (SAPE) and anti-streptavidin antibody (Affymetrix Fluidics Station 400), microarrays were scanned (Affymetrix 7G) and results (Affymetrix GCOS 1.4) were transferred to the NIHLIMS database. Raw data and processed data are available through the National Center for Biotechnology Information Gene Expression Omnibus database (Accession no. GSE16193) and all data is MIAME compliant.

### Quantification of Cytokines

THP-1 cells (0.5×10^6^) or elutriated primary human monocytes (1×10^6^) were incubated with or without LPS (1 µg/ml) in presence or absence of CO (250 ppm) for indicated times. Culture supernatants were collected at the indicated time points and the production of IL-1β and IL-8 was analyzed with Quantikine ELISA kits purchased from R&D system following the manufacturer's instructions.

### Nuclear Run-On Assay

THP-1 cells (2×10^7^) were treated with or without LPS (1 µg/µL) in the presence or absence of 250 ppm CO for various times. Nuclei were isolated with Nuclei Isolation kit (Sigma). *In vitro* reverse transcriptase reactions were carried out at 30°C for 40 min in 250 µL of transcription buffer containing nuclei. Total RNA was extracted (Qiagen) and newly synthesized RNA transcripts were isolated using a µMACS Streptavidin kit (Miltenyi Biotech, Aubum, CA) as previously described [33]. Nascent IL-1β transcripts were quantified by qRT-PCR and normalized to GAPDH.

### Quantitative Real-Time PCR (qRT-PCR)

THP-1 cells (2×10^6^) or elutriated primary human monocytes (3×10^6^) were incubated with or without LPS (1 µg/ml) in presence or absence of CO (250 ppm) for indicated times. Total RNA was isolated from treated cells using RNeasy Mini kits (Qiagen). Reverse transcription was performed with RT kits (La-Roche, Basel Switzerland). Transcript levels of selected genes were measured by qRT-PCR using TaqMan® Universal PCR master mix and the ABI Prism 7900 sequence detection system (Applied Biosystems, Foster City, CA). The gene specific primers and probes for qRT-PCR were purchased from Applied Biosystems. Target gene mRNA levels were normalized to the housekeeping gene GAPDH and relative gene expression was calculated as fold-induction compared with control [34].

### Western Blotting

CO effects on MAPK pathways and IκBα in LPS-stimulated THP-1 cells (2×10^6^) or elutriated primary human monocytes (8×10^6^) were determined by Western blotting. Cells were rinsed with cold PBS, and sonicated in 40 µl of mammalian protein extraction reagent (Pierce) with Complete Mini Protease inhibitor cocktail (Roche, Nutley, NJ). Protein concentrations were estimated by BCA method (Pierce). Proteins (40 µg) were separated on 4–20% SDS Tris-glycine gel (Invitrogen) in 1x SDS Tris-Glycine running buffer, transferred to membranes, blocked with 2% ECL Advance blocking reagent (Amersham Biosciences, Pittsburg, PA) for 1 h at room temperature, and incubated overnight with primary antibody at 4°C followed by appropriate peroxidase-conjugated secondary antibody for 1 h at room temperature. Signal was developed using ECL Advance Western blotting detection kit (Amersham Biosciences) and analyzed using the Image Station 440 charge-coupled device camera system (Eastman Kodak Co., New Haven, CT, USA). Membranes were subsequently stripped using standard stripping solution (100 mM 2-mercaptoethanol, 2% SDS and 62.5 mM Tris.HCl, pH 6.8) at 37°C for 30 min and reprobed with antibody targeting total p38 or α-Tubulin to confirm equal loading of samples.

### Electrophoretic Mobility Shift Assay (EMSA)

Nuclear protein extracts from THP-1 cells (1×10^7^ ) or elutriated primary human monocytes (2×10^7^) treated with and without LPS (1 µg/ml) in the presence or absence of CO (250 ppm) were prepared using CelLytic Nuclear Extraction kit (Sigma, St Louis, MO), according to the manufacturer's recommendations. Double stranded DNA oligonucleotides containing NF-κB consensus sequence (GGGGACTTTCCC) was labeled using Biotin 3′-End DNA Labeling kit (Pierce, Rockford, IL). NF-κB binding activity was determined using the Light Shift Chemiluminesencent EMSA kit (Pierce, Rockford, IL). Nuclear protein extract (5–10 µg) was incubated for 40 min at room temperature in binding buffer containing 200 ng poly dI:dC, 1% NP-40 and 50% glycerol with labeled probe. For competition assays, 100-fold excess of unlabeled NF-κB probe or its mutant probe was added 20 min prior to labeled probe. For supershift assay, 2 µg of rabbit polyclonal antibody against NF-κB p65 or p50 (Santa Cruz Biotechnology) were incubated with nuclear extract 20 min prior to adding labeled probe; likewise antibody against Ah Receptor (C-18) was used as a negative control for NF-κB supershift. Protein-DNA complexes were separated by electrophoresis on 4–20% Tris-Borate-EDTA (TBE) native polyacrylamide gels (Invitrogen) containing 0.5x Tris-Borate-EDTA running buffer at 4°C. Chemiluminescence was measured using the Image Station 440 charge-coupled device camera system (Eastman Kodak).

### Statistical Analysis

Data are presented as mean ± SEM of at least three independent measurements. Fold changes in Table 1 are expressed as point estimates with a confidence interval of one standard error. Messenger RNA and protein expression levels were analyzed by analysis of variance (ANOVA) using experiment as a blocking factor to control for experiment-to-experiment variability. Direct group comparisons of particular interest were examined by *post hoc* least squares means contrasts or paired t-tests. Two- and three-factor ANOVAs were implemented when direct tests of interacting factors were necessary. Differences were considered statistically significant at the α = 0.05 level.

**Table 1:** Validation of microarray results by qRT-PCR in THP-1 cells and primary monocytes.

Gene Symbol	Gene Name	Entrez ID	Fold Change (LPS+CO/LPS)†		
			THP-1 (Microarray)	THP-1 (qRT-PCR)	Primary Monocytes (qRT-PCR)
IL1B	interleukin 1, beta	3553	0.35 (0.30–0.40)	0.48 (0.38–0.59)	0.66 (0.54–0.81)
IL8	interleukin 8	3576	0.71 (0.62–0.81)	0.75 (0.68–0.82)	0.73 (0.61–0.88)
ICAM1	intercellular adhesion molecule 1	3383	0.63 (0.58–0.68)	0.70 (0.65–0.76)	0.49 (0.42–0.58)
CXCL1*	chemokine (C-X-C motif) ligand 1 (melanoma growth stimulating activity, alpha)	2919	0.61 (0.54–0.70)	0.71 (0.66–0.76)	0.51 (0.43–0.61)
CXCL2	chemokine (C-X-C motif) ligand 2	2920	0.62 (0.55–0.70)	0.51 (0.44–0.58)	0.66 (0.57–0.78)
CXCL3*	chemokine (C-X-C motif) ligand 3	2921	0.48 (0.43–0.54)	0.62 (0.53–0.72)	0.50 (0.44–0.56)
CCL3*	chemokine (C-C motif) ligand 3 /// chemokine (C-C motif) ligand 3-like 1 )	414062	0.62 (0.53–0.73)	0.60 (0.45–0.80)	0.69 (0.60–0.78)
CCL4	chemokine (C-C motif) ligand 4	6351	0.38 (0.30–0.46)	0.55 (0.43–0.70)	0.58 (0.39–0.87)
CCL5	chemokine (C-C motif) ligand 5	6352	0.57 (0.51–0.64)	0.57 (0.48–0.68)	0.68 (0.57–0.80)
CCL20	chemokine (C-C motif) ligand 20	6364	0.43 (0.36–0.52)	0.55 (0.48–0.62)	0.42 (0.27–0.66)
EGR2*	early growth response 2	1959	0.35 (0.30–0.41)	0.44 (0.35–0.56)	0.81 (0.76–0.86)
EGR3*	early growth response 3	1960	0.45 (0.38–0.53)	0.51 (0.38–0.68)	0.61 (0.45–0.82)
EGR4*	early growth response 4	1961	0.58 (0.50–0.67)	0.55 (0.42–0.72)	0.33 (0.22–0.48)
PTGS2	prostaglandin-endoperoxide synthase 2	5743	0.47 (0.41–0.54)	0.46 (0.36–0.58)	0.55 (0.45–0.68)
PTX3*	pentraxin-related gene, induced by IL-1 beta	5806	0.58 (0.53–0.65)	0.70 (0.55–0.89)	0.71 (0.64–0.79)
TNF	tumor necrosis factor	7124	0.52 (0.46–0.59)	0.72 (0.64–0.81)	0.74 (0.69–0.79)
TNFAIP3*	tumor necrosis factor, alpha-induced protein 3	7128	0.58 (0.50–0.68)	0.67 (0.52–0.87)	0.57 (0.50–0.66)
ATF3	activating transcription factor 3	467	0.67 (0.59–0.75)	0.87 (0.72–1.05)	0.76 (0.68–0.86)
NFKBIA	nuclear factor of kappa light polypeptide gene enhancer in B-cells inhibitor, alpha	4792	0.82 (0.71–0.93)	0.54 (0.42–0.69)	1.07 (0.99–1.15)
PDE4B*	phosphodiesterase 4B, cAMP-specific	5142	0.60 (0.56–0.64)	0.69 (0.64–0.75)	0.62 (0.48–0.80)

* *New candidate genes for CO suppression.*

† *One standard error confidence interval shown in parentheses.*

For microarray data, expression intensities for each probeset were computed as MAS5 signal intensities, and subsequently quantile-normalized and transformed to homogeneous variance using the “S10” transform available in the MSCL Analyst's Toolbox (http://abs.cit.nih.gov/MSCLtoolbox/). ANOVA was carried out on the transformed values, accounting for batch (factor 1) and 4 distinct treatments (factor 2). The empirical Bayes method of pooling residual variance across many probesets was employed to increase the power of the ANOVA, as implemented in JMP-Genomics (SAS, Inc, Cary, NC). Post-hoc contrasts of LPS vs. control and LPS-CO vs. LPS were computed. A composite p-value for the event that both LPS increased and LPS-CO subsequently decreased the expression levels, was computed as follows: p-both events  =  (max (p_1_,p_2_))^2^, where p_1_ and p_2_ are the calculated p-values for each separate event, and independence of the events is assumed. This approximate p-value was then adjusted for multiple comparisons using the Benjamini-Hochberg approach to false discovery rate (FDR) [35].

Selection criteria for LPS-induced genes at 1 h were FDR ≤10% and fold change (FC) ≥2, which returned 169 probesets representing 101 genes. Gene selection cutoffs for CO-suppression were chosen to capture a large enough list of genes for meaningful bioinformatic analyses and thereby allow us to explore mechanisms underlying this response. A review of the literature and preliminary PCR testing of known CO-target genes demonstrated CO to have modest, but reproducible effects on important inflammatory response genes at the very early time point of interest. These considerations led to the use of liberal criteria for CO-suppression, namely FDR ≤20% and ≥1.2 fold downregulation (corresponding to FC ≤0.83), which returned 79 genes.

Transcription factors were identified among differentially expressed genes using Genomatix® Bibliosphere (Munich, Germany), a text mining database derived from published articles. One additional transcription factor was found using PubMed. NF-κB target genes were similarly first sought using Genomatix® Bibliosphere. Gene promoters from our entire list of CO-suppressed transcripts were also subjected to a sequence-based analysis using Biobase (http://www.biobase-international.com) to identify additional genes with putative NF-κB binding sites. Manual searching of PubMed citations retrieved two NF- κB target genes that were not found by the other methods.

## Results

### Microarrays

**Figure 1:**
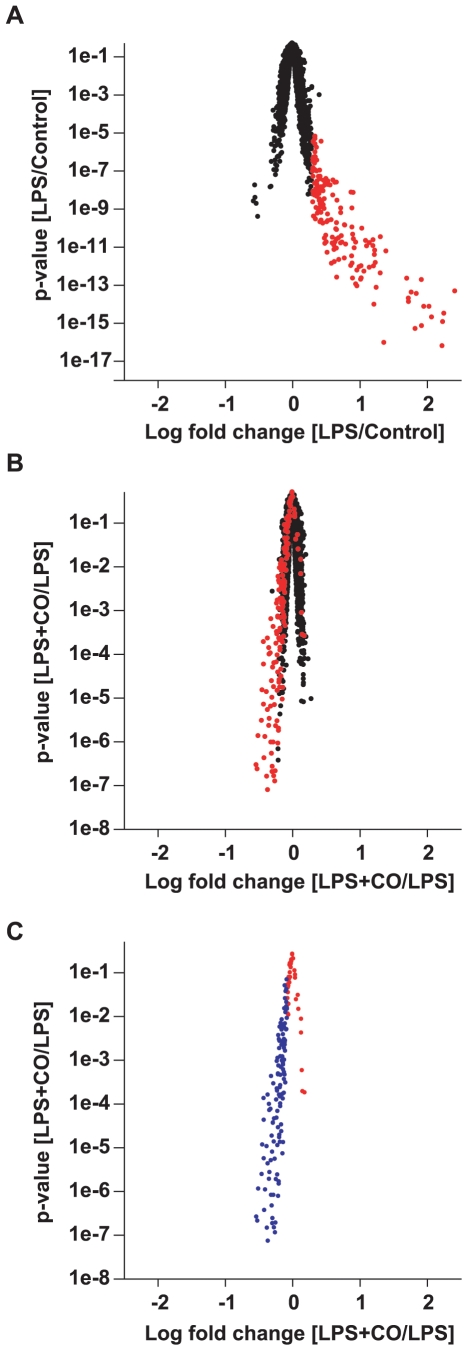
Volcano plot of changes in gene expression. (A) LPS effect compared to control; probesets meeting criteria for LPS upregulation are shown in red. (B) CO effect on LPS exposed cells; again, probesets upregulated by LPS are shown in red. (C) CO effect on the subset of genes that were upregulated by LPS; probesets meeting criteria for CO suppression are shown in blue. THP-1 cells were treated with or without LPS (1 µg/ml) in the presence or absence of CO (250 ppm) for 1 h. LPS-induced genes were selected based on FDR ≤10% and fold change ≥2. CO-suppressed genes required FDR ≤20% and fold change ≤0.83. Some genes are represented by more than one probe on these plots. Raw p-values for the comparison of interest are plotted on the y-axes. Fold changes (x-axes) are the log mean ± SEM of four independent experiments.

Oligonucleotide microarrays identified a genome-wide set of LPS-induced genes that were suppressed by CO at 1 h in THP-1 cells, a human monocytic cell line. In the absence of LPS, CO had little or no effect on gene expression. Only two genes of questionable relevance (putative binding protein 7a5 and uncharacterized gastric protein ZG33P) were identified as CO-suppressed in the absence of LPS, using liberal selection criteria (FDR ≤20% and FC ≤0.83). At 1 h, LPS primarily upregulated gene expression as illustrated by the marked asymmetry of Figure 1A. Likewise, the predominant effect of CO was to suppress these LPS-induced genes (Figure 1B). Of 101 genes (162 probesets) induced by LPS (FDR ≤10%, FC ≥2), CO suppressed the expression of 78% (79 unique genes, 120 probesets), using a liberal selection criteria (FDR ≤20% and FC ≤0.83) to avoid missing important CO effects (Figure 1C). Notably, neither of the two genes that were marginally suppressed by CO in the absence of LPS were significantly suppressed in the presence of LPS. Of LPS-induced genes suppressed by CO, 19% (15/79) were known transcription factors including several EGR family members (Table S1). Inflammatory response genes such as cytokines (IL-1β and TNF-α), chemokines (macrophage inflammatory protein 3 alpha [MIP-3α or CCL20], regulated upon activation, normal T-cell expressed and secreted [RANTES or CCL5], chemokine ligand -1 [CXCL1], CXCL3 and CXCL8 [IL-8]), and inflammatory pathway enzymes (prostaglandin-endoperoxide synthase-2 [PTGS2 or COX2] and phosphodiesterase 4B [PDE4B]) were also prominent among CO-suppressed transcripts (see Table S1 for a complete list). Some of these genes had not been previously identified as targets of CO suppression, including EGR3, EGR4, CXCL1, CXCL3, CCL3, CCL20, pentraxin-related gene (PTX3), TNF alpha-induced protein 3 (TNFAIP3), activating transcription factor 3 (ATF3), nuclear factor of kappa light chain gene enhancer in B-cells (NFKBIA or IκB-alpha), micro RNA-155 (miR-155) and PDE4B. See Table S1 for all newly identified CO-suppressed genes.

### Validation of CO Effects in THP-1 Cells and Primary Human Monocytes

Twenty CO-suppressed transcripts identified by microarray were confirmed in THP-1 cells using qRT-PCR. Elutriated monocytes from normal volunteers were also studied by qRT-PCR to determine whether CO suppressed a similar set of LPS-induced genes in primary cells. For THP-1 cells, qRT-PCR compared to microarray results was directionally concordant for all 20 genes. Nineteen of 20 transcripts independently met criteria as significantly CO-suppressed by qRT-PCR (p<0.05, ANOVA *post hoc* contrast of LPS+CO *vs.* LPS; Table 1). Like THP-1 cells, elutriated primary human monocytes were challenged with LPS for 1 h in the presence or absence of CO gas (250 ppm). For primary human monocytes, 19 of these 20 genes were directionally concordant with the microarray results in THP-1 cells and these same 19 were individually confirmed as significantly CO-suppressed (p<0.05, ANOVA *post hoc* contrast of LPS+CO *vs.* LPS; Table 1).

**Figure 2:**
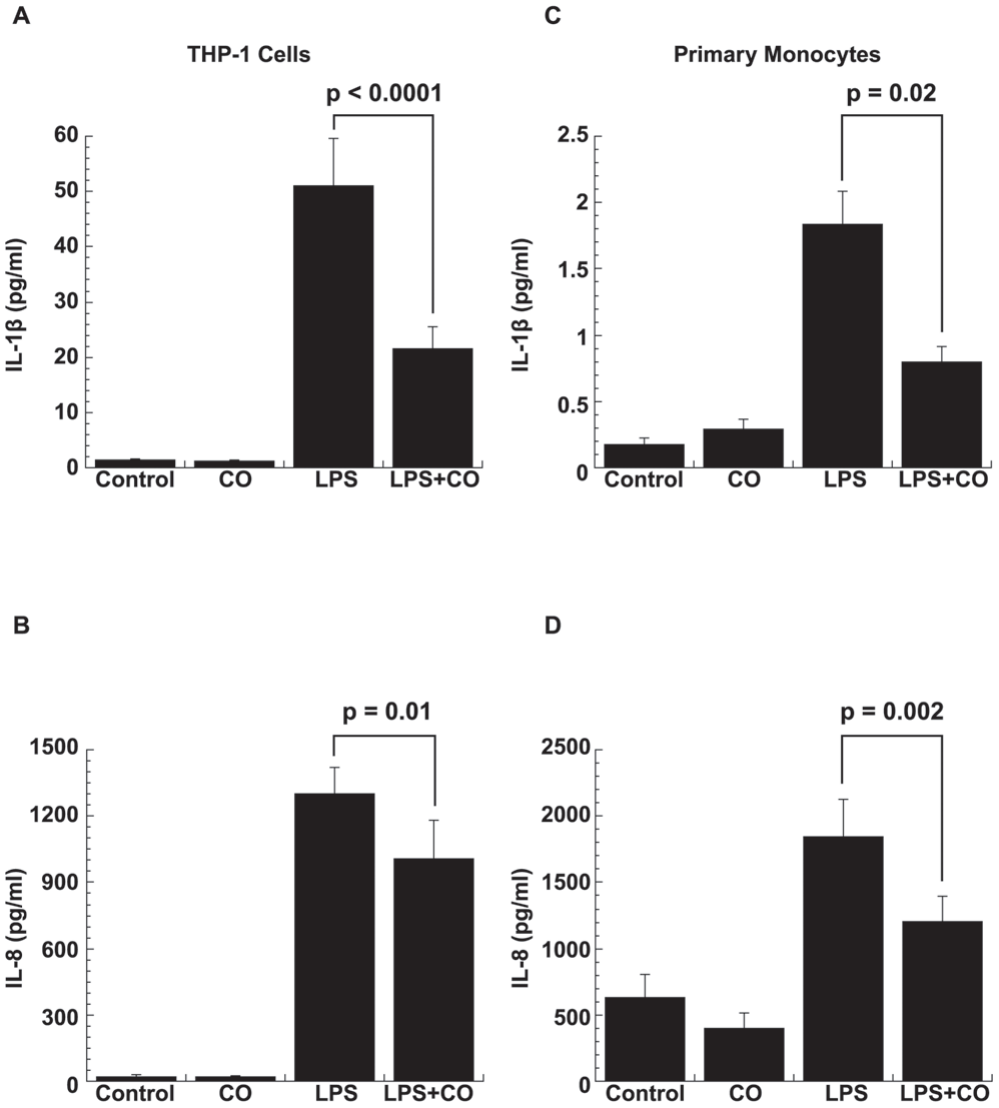
CO downregulation of LPS-induced IL-1β and IL-8 protein. CO downregulated LPS-induced production of (A) IL-1β and (B) IL-8 in THP-1 cells (p<0.02 for both), and likewise (C) IL-1β and (D) IL-8 in primary human monocytes (p ≤0.02 for both). THP-1 cells (0.5×10^6^) or primary human monocytes (1×10^6^) were incubated with LPS (1 µg/ml) in the absence or presence of CO (250 ppm) for 2 h. Cytokine production was measured in supernatants by ELISA. Results are mean ± SEM of at least four independent experiments for THP-1 cells and seven healthy blood donors for primary monocytes.

For validation at the protein level, two secreted genes (IL-1β and IL-8) were chosen for further evaluation. As expected, treatment with LPS (1 µg/ml) increased IL-1β and IL-8 production at 2 h and exposure to CO (250 ppm) significantly down-regulated this response in both THP-1 cells (p<0.02 for both, paired t-test; Figure 2, A and B, respectively) and primary human monocytes (p<0.02 for both, paired t-test; Figure 2, C and D, respectively).

### Time Course of CO Suppression

**Figure 3:**
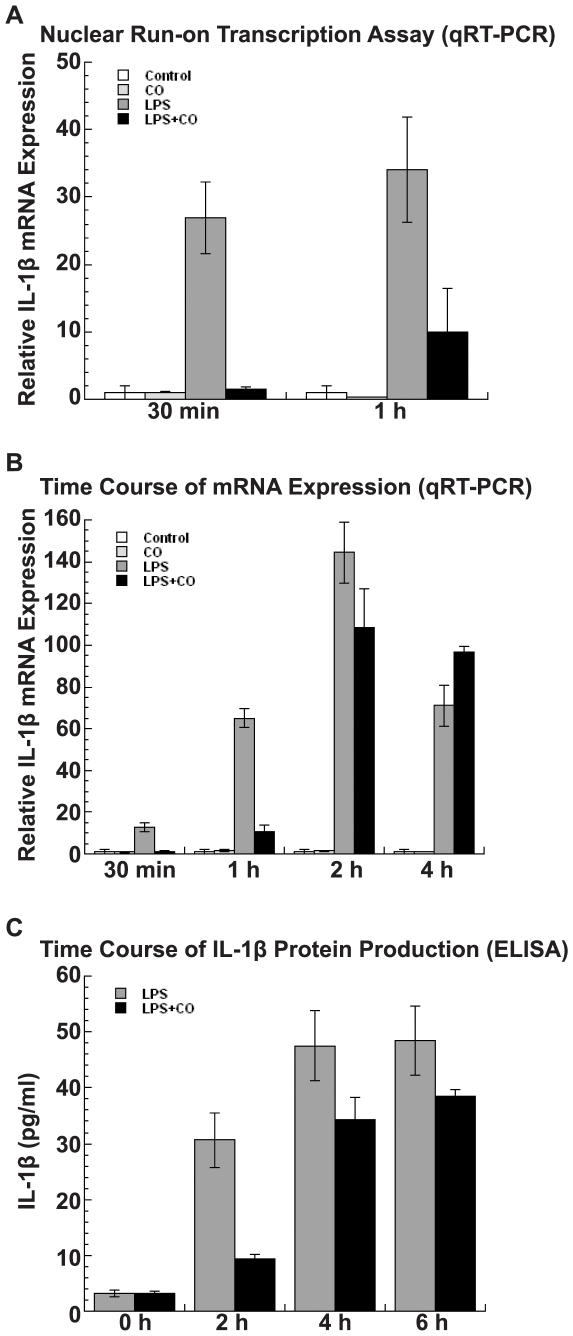
CO suppression of LPS-induced IL-1β transcription, mRNA expression, and protein production. (A) Nuclear run-on assays in THP-1 cells (2×10^7^) showed that CO represses IL-1β transcription by almost 98% within 30 min of LPS stimulation (p = 0.001) and the effect decreased to 67% at 60 min (p = 0.01). (B) A time course analysis of IL-1β mRNA expression in THP-1 cells (2×10^6^) by qRT-PCR showed that CO strongly suppressed LPS-induced IL-1β mRNA by more than 90% within 30 min of exposure (p<0.0001), an effect that was no longer present at 4 h. (C) Time course analysis of IL-1β protein production in THP-1 cells (0.5×10^6^) showed maximum suppression of CO on LPS-induced IL-1β at 2 h (p = 0.04). Results are mean ± SEM of three independent experiments.

Next, a time course of IL-1β transcription and protein expression was evaluated in THP-1 cells using qRT-PCR. CO suppression of IL-1β, a prototypic inflammatory response gene, was found to be both early and transient. Nuclear run-on assays demonstrated that CO suppressed IL-1β transcription by 98% at 30 min after LPS stimulation (p = 0.001), and by 67% at 1 h (p = 0.01, ANOVA *post hoc* contrast of LPS+CO *vs.* LPS; Figure 3A). Consistent with these effects on transcription, CO suppressed LPS-induced IL-1β mRNA expression by 96% at 30 min, (p<0.0001; Figure 3B). Again, the size of this effect decreased over time and CO no longer suppressed IL-1β mRNA 4 h after LPS exposure. Similarly, a time course of IL-1β protein secretion showed a reduction in the CO effect from 69% suppression at 2 h to only 20% at 6 h (p = 0.1, Cochran-Armitage test of linear trend; Figure 3C).

### CO Effects on Stress Kinase Pathways in THP-1 Cells

**Figure 4:**
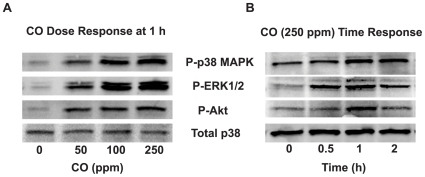
CO activation of stress kinases. THP-1 cells (2×10^6^) were treated (A) with different doses of CO gas (0–250 ppm) for 1 h or (B) with 250 ppm CO gas for various time points (0–2 h). Whole cell lysates (40 µg) were examined for phosphorylated p38 MAPK, ERK1/2 and Akt by Western blotting. Total p38 MAPK showed equal loading and transfer of protein. Results shown are representative blots from three independent experiments.

**Figure 5:**
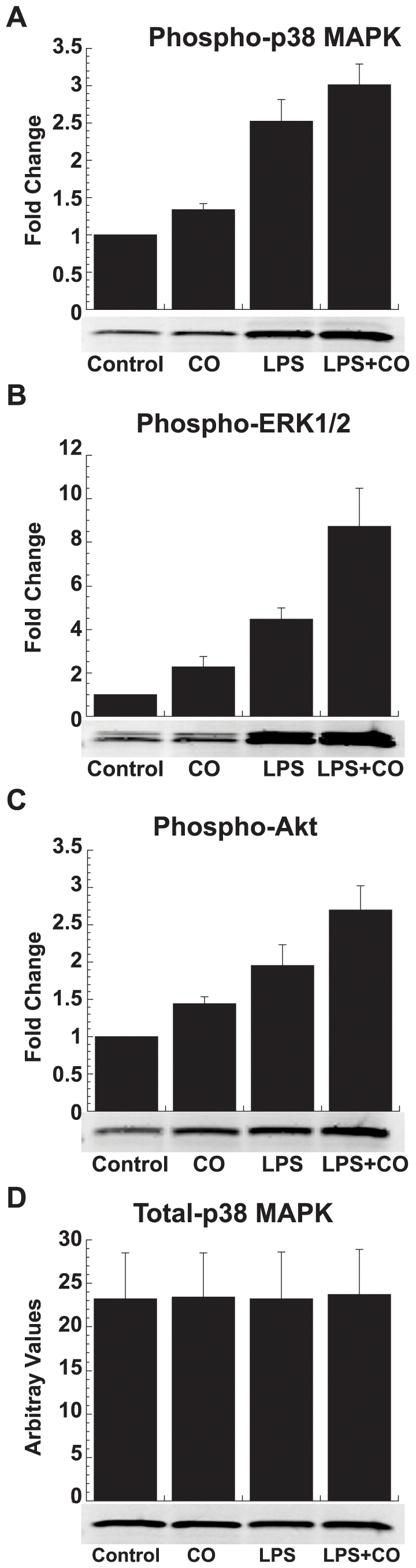
CO activation of LPS-induced stress kinases. CO and LPS activated (A) p38 MAPK, (B) ERK1/2, and (C) Akt as measured by Western blotting. (D) Total p38 served as a loading control. CO and LPS effects for all three kinases were additive. THP-1 cells (2×10^6^) were treated with or without LPS (1 µg/ml) in the presence or absence of CO gas (250 ppm) for 1 h. Densitometric analysis is plotted as fold change from control. Results shown are mean ± SEM of three independent experiments along with representative blots.

**Figure 6:**
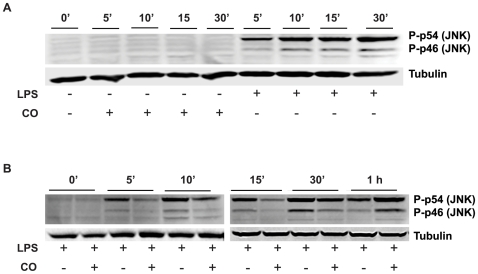
CO effects on LPS-induced JNK activation. (A) Effects of CO and LPS alone on JNK activation. (B) Time-kinetics of LPS (1 µg/ml) induced JNK activation evaluated in the presence or absence of CO (250 ppm). Zero to 10 min and 15 min to 1 h were run at the same time, but on separate gels. Whole cell lysates (40 µg) from THP-1 cells (2×10^6^) were examined for phosphorylated JNK by Western blotting. α-Tubulin demonstrated equal loading and transfer of protein. Results shown are representative blots from at least two independent experiments.

The immediate onset and rapid decay of CO suppression was similar in pattern to the manner in which LPS-TLR4 signaling activates major stress kinase pathways. Given their importance in LPS-induced gene expression and previous reports linking CO suppression of inflammation to these pathways, the modulation of stress kinases was explored experimentally. CO activated p38 MAPK, pERK1/2 and Akt at 30 min as determined by phosphorylation in a dose (Figure 4A) and time (Figure 4B) dependent manner. CO main effects (p<0.02 for all) were additive with those of LPS for each kinase (p<0.001 for an LPS main effect; p>0.12 for a LPS*CO interaction; Figure 5, A–C). In contrast, CO exposure (250 ppm) did not directly activate JNK (Figure 6A), but rather delayed the early phase of LPS-induced JNK phosphorylation in THP-1 cells (Figure 6B). At 1 h, LPS-induced JNK activation in the presence of CO reached the same intensity as the maximum effect of LPS alone (seen at 30 min), even as the LPS-only response began to wane.

**Figure 7:**
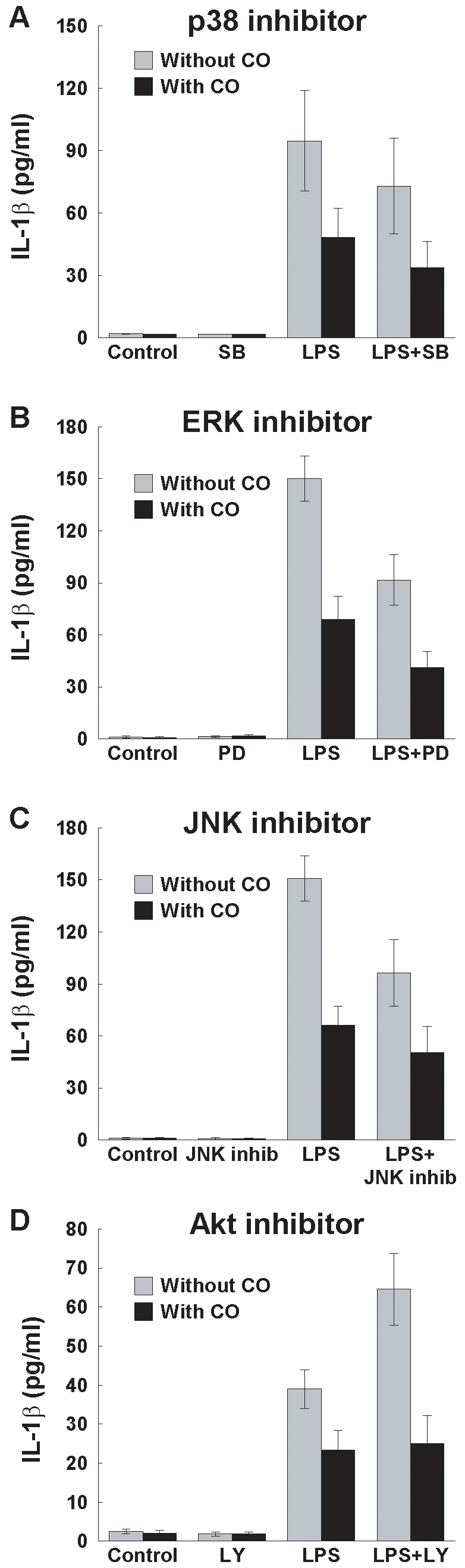
Effects of CO on LPS-induced IL-1β production in the presence or absence of stress kinase inhibitors. THP-1 cells (2×10^6^) were pretreated with inhibitors of (A) p38 MAPK (SB202190; 0.1 µM), (B) ERK1/2 (PD98059; 30 µM), (C) JNK (Inhibitor II; 10 µM), and (D) Akt (LY294002; 10 µM) for 30 min and then stimulated with LPS (1 µg/ml) in the presence or absence of CO gas (250 ppm) for 2 h. DMSO (0.1%) vehicle was used as control. IL-1β production was measured in supernatants by ELISA. Results are mean ± SEM of three to five independent experiments.

Despite these effects of CO on kinase pathways, CO suppressed LPS induction of IL-1β in the absence (p≤0.04 for all) and presence (p≤0.055 for all) of specific kinase inhibitors (Figure 7, A–D). Inhibitors of p38 MAPK, ERK1/2, and JNK all failed to prevent or significantly decrease CO suppression of IL-1β induction by LPS (p≥0.18 for an interaction between CO suppression and any of these three inhibitors; Figure 7, A, B and C). Interestingly, Akt inhibition increased LPS induction of IL-1β, while CO suppression became proportionally larger (p = 0.04 for an interaction between CO and the Akt inhibitor; Figure 7D). Akt inhibitor-mediated increases in CO suppression suggest that CO activation of Akt may actually interfere slightly with CO suppression of inflammation. Collectively, inhibitor experiments provided no evidence indicating that any of these kinase pathways are primarily responsible for CO suppression of inflammation.

### Association of CO Suppression with NF-κB Signaling

In addition to stress kinase pathways, LPS-TLR4 signal transduction leads to MyD88-dependent, early phase and MyD88-independent late phase activation of NF-κB. Ingenuity Pathway® analysis identified the canonical NF-κB pathway as significantly over-represented in our microarray list of CO-suppressed genes (p<0.001). Of 79 immediate-early genes suppressed by CO, 67% (53/79) had NF-κB binding sites in their promoters based on an analysis using Genomatix® Bibliosphere. A sequence-based promoter analysis of our CO-suppressed genelist using Biobase (http://www.biobase-international.com) found that 54% (43/79) had putative NF-κB binding sites (p≤0.03; hypergeometric test comparing the genelist vs. 562 human housekeeping genes). Manual searches of PubMed found another five NF-κB regulated genes not recognized by Genomatix®, two of which were also not identified to have NF-κB binding sites by Biobase. Overall 81% (64/79) of CO-suppressed genes were NF-κB targets as determined by one or more of the above approaches (Table S1).

**Figure 8:**
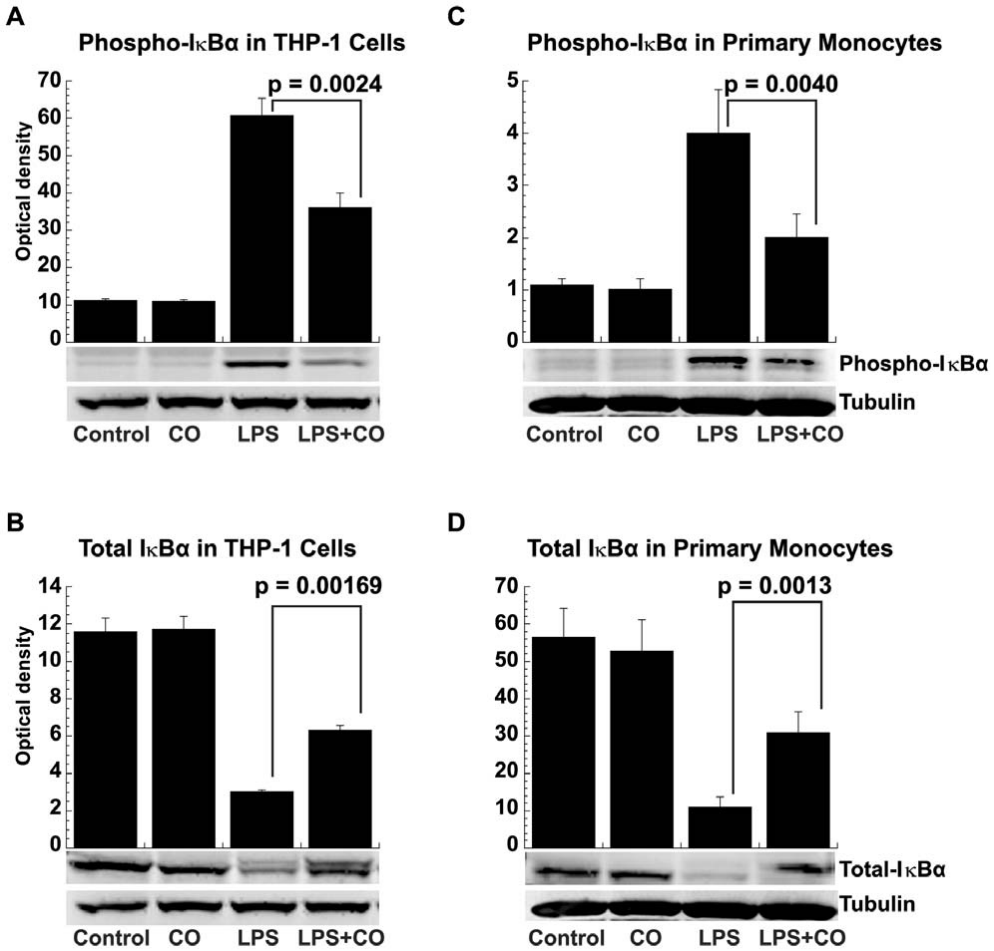
CO prevention of LPS-induced IκBα phosphorylation and degradation. (A) Phosphorylated IκBα in THP-1 cells, (B) total IκBα in THP-1 cells, (C) phosphorylated IκBα in primary human monocytes, and (D) Total IκBα in primary human monocytes. Whole cell extracts or cytoplasmic fractions were prepared from THP-1 cells (2×10^6^) and elutriated primary human monocytes (5×10^6^), treated with or without LPS (1 µg/ml) in the presence or absence of CO (250 ppm) for 15 min and examined for phosphorylated or total IκBα by Western blotting. α-Tubulin served as a equal loading and transfer control. Densitometry results are shown as the means ± SEM of three independent experiments.

Both THP-1 cells and primary human monocytes were used to directly test the association of CO suppression with NF-κB signaling as suggested by the microarray results. CO was found to block an early step in LPS-induced NF-κB activation. Cytoplasmic and total cell protein extracts were analyzed by Western blotting for total IκBα and IκBα phosphorylated at serine residues 32 and 36. LPS exposure induced the rapid (within 15 min) phosphorylation and degradation of IκBα in both THP-1 cells (p<0.0001 for both processes; Figure 8, A and B) and elutriated primary human monocytes (p = 0.003 and p<0.0001, respectively, ANOVA *post hoc* contrast of LPS *vs.* control for each; Figure 8, C and D). Conversely, CO inhibited LPS-induced IκBα phosphorylation (p = 0.002) and degradation (p = 0.004) in THP-1 cells (Figure 8, A and B, respectively). Similar effects of CO on IκBα phosphorylation (p = 0.016) and degradation (p = 0.001) were also documented in primary human monocytes (Figure 8, C and D, respectively), showing that this mechanism of action was not limited to an idiosyncratic cell line.

**Figure 9:**
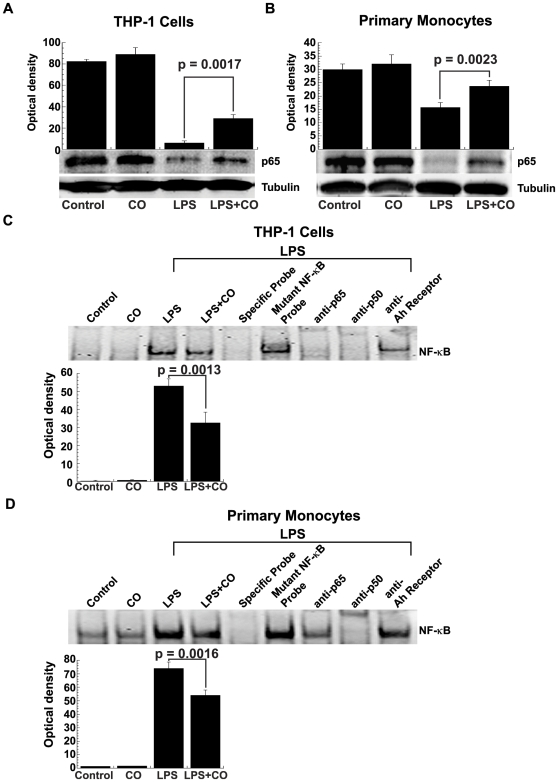
CO inhibition of LPS-induced NF-κB nuclear translocation and activation. CO inhibited LPS-induced nuclear translocation of NF-κB from the cytoplasm (A) THP-1 and (B) primary human monocytes as shown by Western blotting using antibodies against p65. Cytoplasmic fractions were made by treating the cells with or without LPS (1 µg/ml) in presence or absence of CO (250 ppm) for 30 min. Nuclear extracts were prepared to measure NF-κB DNA binding by EMSA in (C) THP-1 cells and (D) primary human monocytes using NF-κB probe. Supershift of the LPS-induced NF-κB complex is shown using anti-p65 and anti-p50 antibodies, while anti-Ah Receptor (C-18) antibody served as a negative control. THP-1 cells (1×10^7^) and primary human monocytes (2×10^7^) were stimulated with or without LPS (1 µg/ml) in the presence or absence of CO (250 ppm) for 30 min. Densitometry results are shown as the means ± SEM of three independent experiments. Results are representative gels from at least three experiments.

IκBα phosphorylation and degradation frees NF-κB from sequestration, allowing it to translocate to the nucleus, bind to target promoters, and turn-on transcription. LPS stimulation caused NF-κB p65 translocation from the cytoplasm to the nucleus while CO interfered with this effect in THP-1 cells (p = 0.002; Figure 9A) and primary human monocytes (p = 0.002; Figure 9B), as seen by Western blotting.

EMSA were performed using nuclear extracts from THP-1 cells and primary human monocytes to further demonstrate CO effects on NF-κB signaling. As expected, LPS increased the formation of a protein complex with NF-κB consensus sequence probe. CO exposure reproducibly reduced NF-κB complex formation in both THP-1 cells and primary human monocytes (p = 0.001 and p = 0.002; Figure 9, C and D, respectively). Experiments were carried out with 100-fold molar excesses of unlabeled specific probe that competed with NF-κB binding and mutant probe that did not, demonstrating the sequence specificity of NF-κB bands. Finally, anti-p65 and anti-p50 antibody super-shifted and/or diminished the density of NF-κB-protein complexes in both THP-1 cells and primary human monocytes (Figure 9, C and D, respectively) confirming the presence of these proteins. An irrelevant anti-Ah Receptor (C-18) antibody, used as negative control, did not alter the LPS-induced NF-κB complexes (Figure 9, C and D, respectively).

## Discussion

CO has been linked to vascular health and identified as a cytoprotective messenger in lung injury. This has led to speculation about the potential therapeutic benefits of CO in septic shock and pulmonary inflammation. However, most mechanistic and therapeutic research on CO has been performed in rodent models of inflammation and tissue injury. This has importantly advanced several mechanisms of CO action, identified a number of downstream gene targets, and shown beneficial effects of CO in models of ischemia reperfusion injury [13], transplant rejection [36], ventilator-induced lung injury [37] and septic shock [7], [38]. Exploration of CO signaling in human cells suggests extensive overlap with findings in rodents, yet a recent study in endotoxin challenged volunteers failed to detect clinically important anti-inflammatory effects *in vivo* [32].

Here microarrays were used to more globally characterize the effects of CO on the early LPS- induced inflammatory response of human cells. Although conducted in THP-1 cells, a monocytic cell line, many CO-suppressed genes were subsequently confirmed in primary human monocytes. CO was found to broadly suppress LPS-induced immediate-early genes that initiate and propagate inflammation including pivotal transcription factors (e.g. EGR family members and ATF3), cytokines (e.g. IL-1β and TNF-α) and chemokines (CCL5, CXCL1 and IL-8). Using IL-1β as a prototypic target gene, CO suppression of transcription was found to be very fast, but transient, a response pattern reminiscent of LPS-induced kinase activation. While CO activated p38 MAPK, ERK1/2 and Akt and perhaps more importantly delayed LPS-induced JNK activation, inhibitors of these kinase pathways failed to substantially block CO suppression as measured by effects on IL-1β. An analysis of CO suppressed genes revealed that most were transcriptionally regulated by NF-κB. Like the stress kinase pathways, NF-κB is a target of MyD88-dependent early phase LPS-TLR4 signaling. CO was subsequently shown to significantly block an early step in LPS-induced NF-κB activation. Collectively our findings support a mechanism for the anti-inflammatory actions of CO that targets a proximal step in LPS-TLR4 signal transduction, before the divergence of the JNK and NF-κB pathways.

The genome-wide effects of CO on gene expression have not been previously investigated in human monocytes. Our experimental design focused on early gene regulation downstream from LPS-TLR4 signal transduction. Using this approach many genes previously reported to be CO responsive either in mouse or human cells were also identified here such as TNF-α [7], [37], MIP-β [7], IL-1β [7], [18], IL-8 [21], ICAM-1 [16] and COX-2 [39]. In addition to these known CO target genes, new CO-responsive transcripts were detected for the first time by microarray including CCL3, CXCL1, CXCL3, PTX3, PDE4B, NFKBIA, ATF3, EGR family members other than EGR1, miR-155 and TNFAIP3. CO suppression of numerous chemokines beyond those initially described indicates an ability of CO to broadly modulate chemotaxis and cell recruitment, early events in establishing a local inflammatory response. PTX3, another novel CO target, is produced at sites of infection by macrophages and has been implicated in acute lung injury [40]. PDE4B, also newly discovered here as a CO-suppressed gene is the only known LPS-inducible PDE4 subtype and ablation of this gene protects mice from LPS-induced shock [41]. EGR family members are zinc-finger transcription factors associated with ischemia/reperfusion injury [17]. While EGR-1 was already a recognized CO-target, the finding that CO-suppresses multiple family members suggests a more general role in regulating vascular inflammation due to ischemia [26], [42].

In addition to decreasing the expression of proinflammatory genes, CO also suppressed several transcripts associated with negative feedback mechanisms that control the intensity of immune responses. LPS-TLR4-NF-κB signaling is known to induce miR-155, a microRNA that acts like a brake on inflammation [43]. CO suppression of miR-155 might therefore intensify the response to LPS. Similarly CO also suppressed TNFAIP3, a proapoptotic protein that terminates TLR-induced NF-κB activition and inflammatory gene induction in macrophages [44]. As such, CO suppression of miR-155 and TNFAIP3 could be proinflammatory and allow early phase inhibition of NF-κB signaling by CO to recover. Interestingly, using IL-1β as a protypic gene, CO suppression was found to be transient. In fact, by 4 h after LPS challenge, IL-1β mRNA levels were slightly higher in CO exposed cells. The time dependent loss of CO anti-inflammatory effects has not been reported previously. However, this finding may have implications for the therapeutic application of CO in patients.

We found that CO activated p38 MAPK, ERK1/2 and Akt, but delayed LPS-induced JNK activation in human monocytes. Other investigators have previously identified one or more of these stress kinase pathways as central to the anti-inflammatory effects of CO. LPS-challenged murine macrophages or mice [7] preconditioned with low dose CO gas showed reductions in the production of TNF-α, IL-1β and MIP-1β and increases in IL-10 that were mediated by p38 MAPK. Mice deficient in MKK3, an upstream activator of p38 MAPK failed to modulate TNF-α or upregulate IL-10 in response to CO after LPS challenge. In our experiments, CO effects were additive with LPS in activating p38 MAPK, but unlike the mouse experiments, inhibiting p38 MAPK did not block CO suppression of IL-1β. In addition to p38 MAPK activation, CO inhibition of ERK1/2, an effect opposite to our results in human monocytes, has been associated with the suppression of inflammation. In human pulmonary epithelial cells, IL-17 induction of IL-6 was reduced by CO through interference with ERK1/2 activation [29]. Consistent with these findings, another group demonstrated that CO protection from ischemic lung injury in rats was mediated by the inhibition of ERK activation and subsequent EGR-1 induction [17]. Interestingly, CO inhibition of ERK1/2 in rats was cGMP dependent. In our human monocyte study, CO suppression of early LPS-induced genes was cGMP independent (data not shown), ERK1/2 was activated rather than inhibited by CO, and ERK blockade did not alter the CO effect.

Although we found that CO activated Akt in human monocytes, unlike a study of cardiac ischemia/reperfusion injury in rats [45], inhibition of this pathway enhanced rather than attenuated the impact of CO. We like others, found that CO delayed LPS-induced JNK activation. It was shown in mouse macrophages that delay of JNK activation by CO reduced LPS induction of IL-6 through effects at an AP-1 promoter-binding site [18]. CO-mediated delay of JNK activation is consistent with a proximal disruption in LPS-TLR4 signal transduction that could alter the kinetics of gene expression. However, this mechanism was not essential for IL-1β suppression by CO. Notably, CO effects on JNK have also been associated with cytoprotection from apoptosis [46], an important function of CO that was not examined in the current investigation.

An *in silico* analysis of our microarray results suggested that the canonical NF-κB pathway might be a pivotal effector of CO suppression in human monocytes. NF-κB is held dormant in the cytoplasm of resting cells by IκB. LPS challenge leads to the rapid phosphorylation and degradation of IκB, allowing NF-κB to translocate to the nucleus, where it binds to promoters and activates the transcription of inflammatory response genes [44], [47], [48]. A role for NF-κB in CO suppression is supported by previous studies in murine macrophages [19], human umbilical vein endothelial cells [30], and Caco-2 cells [21], a human colonic adenocarcinoma line. CO inhibition of NF-κB activation has been associated with the suppression of a number of genes including GM-CSF [19], iNOS, ICAM-1[16], IL-8, IL-6 and MMP-7 [21]. While CORM-2 was shown to attenuate NF-κB activation in the livers of septic mice, another *in vivo* study of inhaled CO found no effect on NF-κB activation in rat liver allografts [49]. Two other investigations studying the anti-apoptotic activity of CO have reported CO-mediated NF-κB activation in rat hepatocytes [24], and mouse, cow and human endothelial cells [23]. Nonetheless, CO (250 ppm) was shown in our experiments to reproducibly disrupt IκBα phosphorylation and degradation, and subsequent NF-κB activation in LPS-stimulated human monocytes.

Although CO interference with LPS-TLR4 activation of NF-κB appears to be a pivotal event in the early suppression of gene transcription, the exact mechanism and molecular target(s) of this effect are not entirely clear. Immediate events in LPS-TLR4 signal transduction include the recruitment of adaptor proteins such as MyD88. The MyD88-dependent arm of LPS-TLR4 signaling culminates in the rapid-phase activation of NF-κB and stress kinase pathways. In parallel, the MyD88-independent pathway leads to a slightly later phase of NF-κB activation. Nakahira and colleagues have reported in murine RAW264.7 cells compelling results demonstrating that CO disrupts the recruitment of essential adaptor proteins, specifically MyD88 and TRIF, to the TLR4 receptor complex, thereby reshaping LPS signal propagation [20]. Alterations in these very early LPS-TLR4 signaling events seem like a plausible explanation for both the brief delay in JNK activation and interference with NF-κB-mediated gene induction. Given the regulatory complexity, it also seems likely that changes in the kinetics of adaptor protein recruitment might produce a variety of stress kinase pathway effects depending on the specific cellular context.

CO was shown to suppress immediate-early gene induction in LPS challenged human monocytes. CO activated several stress kinase pathways, while LPS-induced JNK activation was delayed and NF-κB signaling was impaired. Future investigations should focus on early events upstream from NF-κB and stress kinase pathway activation, as well as clinical studies to determine the utility of CO-based therapies in the management of acute inflammation. Our results would suggest that the greatest benefits of CO might be realized in situations where the inflammatory challenge is predictable and brief such as cardiac bypass or ischemia/reperfusion in transplant surgery.

## Supporting Information

Table S1 (0.04 MB XLS)
